# Outcomes of Primary 27-Gauge Vitrectomy for 73 Consecutive Cases With Uveitis-Associated Vitreoretinal Disorders

**DOI:** 10.3389/fmed.2021.755816

**Published:** 2021-10-27

**Authors:** Kyung Woo Kim, Sentaro Kusuhara, Hisanori Imai, Noriyuki Sotani, Ryuto Nishisho, Wataru Matsumiya, Makoto Nakamura

**Affiliations:** Division of Ophthalmology, Department of Surgery, Kobe University Graduate School of Medicine, Kobe, Japan

**Keywords:** uveitis, 27-gauge, vitrectomy, visual acuity, inflammation, complication

## Abstract

**Background:** Since the advent of 27-gauge microincision vitrectomy system a decade ago, evidence regarding the feasibility, safety, and effectiveness of 27-gauge pars plana vitrectomy (PPV) has increased.

**Aim:** To assess the effectiveness and safety profile of 27-gauge PPV for various vitreoretinal conditions associated with uveitis.

**Methods:** We retrospectively investigated 73 consecutive cases that underwent primary 27-gauge PPV for uveitis-related ocular disorders between October 2014 and April 2021. The primary outcome measures were mean change in logMAR best-corrected decimal visual acuity (BCVA) pre-operatively to 3 months post-operatively, the proportion of BCVA improvement category defined as the degree of logMAR BCVA difference (“improved” [≤−0.3], “unchanged” [−0.3 to 0.3], and “worsened” [≥0.3]) pre-operatively to 3 months post-operatively, the mean change in intraocular inflammation scores pre-operatively to 3 months post-operatively; and intraoperative and post-operative complications.

**Results:** The mean logMAR BCVA significantly improved from 0.69 pre-operatively to 0.42 at 3 months post-operatively (*P* = 0.017). The percentages of eyes with “improved,” “unchanged,” and “worsened” BCVA at 3 months post-operatively were 37, 50, and 13%, respectively. The mean anterior chamber cell score was 0.6 pre-operatively and 0.2 at 3 months post-operatively (*P* = 0.001), the mean anterior chamber flare score was 0.4 pre-operatively and 0.1 at 3 months post-operatively (*P* = 0.004), and the mean vitreous haze score was 1.9 pre-operatively and 0.1 at 3 months post-operatively (*P* < 0.001). Surgery-related complications occurred in 35 (48%) eyes, 68% of which were related to intraocular pressure and transient.

**Conclusions:** Given its risk–benefit profile, 27-gauge PPV is a promising option for the treatment of vitreoretinal disorders in uveitis.

## Introduction

Medical therapy is the cornerstone for the management of uveitis, a potentially sight-threatening intraocular inflammatory disorder (1, 2). However, pars plana vitrectomy (PPV) may be performed when medical therapy fails, secondary vitreoretinal complications develop, or diagnostic vitreous/retinal sampling is necessary (3–12). The benefits of PPV in eyes with uveitis chiefly stem from the removal of vitreous gel and abnormal tissues that cause media opacification, intraocular accumulation of inflammatory mediators, and/or retinal complications (12). However, the surgical stress associated with PPV increases the risk of intraoperative and post-operative complications in inflamed eyes (7, 12, 13). Therefore, balancing benefits with risks has become the primary concern when performing PPV for uveitis (12, 14, 15).

The microincision vitrectomy system (MIVS), first appeared as 25-gauge PPV, was developed nearly two decades ago (16) and has gradually become the standard surgical platform for various indications, including uveitis-related vitreoretinal disorders (8, 9). Recent technological advances have enabled PPV using 27-gauge instruments (27-gauge PPV) as Oshima and associates documented in 2010 (17). This smaller-gauge instrumentation can enhance the advantages of MIVS, such as reduced patient discomfort, minimal scarring, and rapid recovery of vision post-operatively. Thus far, many researchers have reported the feasibility, safety, and efficiency of 27-gauge PPV (17–24). Given its less invasive nature, 27-gauge PPV should be suitable for vitreoretinal disorders secondary to uveitis. However, to the best of our knowledge, the usefulness of 27-PPV for eyes with uveitis remains unknown. To address this research gap, this study aimed to assess the effectiveness and safety profile of 27-gauge PPV for various vitreoretinal conditions associated with uveitis.

## Materials and Methods

### Study Design and Patients

This single-center retrospective study was approved by the institutional review board of the Kobe University Graduate School of Medicine (permission number: 170115) and adhered to the tenets of the Declaration of Helsinki for research involving human subjects. Informed consent was not obtained from patients due to the retrospective, observational nature of this study. However, patients were able to withdraw their consent anytime through the opt-out choice provided on the hospital homepage.

We reviewed the medical records of 73 consecutive eyes from 73 patients who underwent primary 27-gauge PPV for uveitis-related ocular disorders between October 2014 and April 2021 at Kobe University Hospital. Patients with a history of 27-gauge PPV for the fellow eye were excluded (*n* = 13) to evade inter-eye correlation in statistical analyses. The collected data included the following: age, sex, laterality of the operated eye, anatomical type of uveitis, cause of uveitis, lens status, best-corrected decimal visual acuity (BCVA) (converted to the logarithm of the minimum angle of resolution [logMAR] for analysis), intraocular pressure, anterior chamber cell, anterior chamber flare, vitreous haze, presence or absence of retinal and/or choroidal lesion, purpose of surgery, date of surgery, concomitant cataract surgery, tamponade agent, surgical time, complications, and final visit date. Intraocular inflammation scoring for the anterior chamber cell, anterior chamber flare, and vitreous haze was carried out according to the National Eye Institute criteria adapted by the Standardization of Uveitis Nomenclature Working Group (25).

### Surgical Technique

All surgeries were performed by two experienced surgeons (SK and HI). All patients received local anesthesia consisting of 4% lidocaine eye drops and sub-Tenon injection of ~4 mL of 2% lidocaine or 0.75% ropivacaine hydrochloride. General anesthesia was performed as necessary. Patients' skin and ocular surfaces were disinfected using 5% povidone-iodine and eight-fold diluted PA · IODO Ophthalmic and Eye washing Solution Disinfection (Nitten Pharmaceutical Co., Nagoya, Japan). The Constellation Vision System (Alcon Laboratories, Inc., Fort Worth, TX, USA) combined with a 27+ Combined Procedure Pak (Alcon Laboratories, Inc.) and a wide-angle non-contact viewing system (Resight 500 or 700, Carl Zeiss Meditec AG, Jena, Germany) were used for all surgeries. A 27G Oshima Vivid chandelier (Bausch and Lomb, St. Louis, MO) was utilized for illumination as needed. Combined cataract surgery, phacoemulsification, and intraocular lens implantation were performed using the Constellation Vision System (Alcon Laboratories) as needed. We routinely removed as much vitreous gel as possible irrespective of the purpose of the surgery.

### Outcomes

The primary outcome measures were mean change in logMAR BCVA pre-operatively to 3 months post-operatively, the proportion of BCVA improvement category defined as the degree of logMAR BCVA difference (“improved” [≤−0.3], “unchanged” [−0.3 to 0.3], “worsened” [≥0.3]) pre-operatively to 3 months post-operatively, the mean changes in intraocular inflammation scores pre-operatively to 3 months post-operatively, and intraoperative and post-operative complications.

### Statistical Analyses

All statistical analyses were carried out as complete case analyses; cases with any missing data were excluded from analysis at each time point. The Wilcoxon test was used for time comparisons (baseline and 3 months post-operatively) for each variable. Statistical analyses were performed using MedCalc v.16.8.4 software (MedCalc Software, Belgium). *P*-value < 0.05 was considered statistically significant.

## Results

The pre-operative characteristics of the included patients are summarized in Table 1. The purposes of surgery (number of eyes) were biopsy (38 [52%]), removal of vitreous opacity (12 [16%]), epiretinal membrane peeling (7 [10%]), reduction of infectious pathogens (6 [8%]), repair of retinal detachment (3 [4%]), removal of vitreous hemorrhage (3 [4%]), removal of retained lens fragment (2 [3%]), closure of macular hole (1 [1%]), and improvement of macular edema (1 [1%]). Of the total 39 phakic eyes, 38 (97%) underwent concomitant cataract surgery (phacoemulsification and intraocular lens implantation). Intraocular tamponade was performed in 13 (18%) eyes. The tamponade agents used (number of eyes) were air (5 [38%]), 20% SF_6_ gas (2 [15%]), and silicone oil (6 [46%]). The mean surgical time was 47.1 ± 26.1 min and the mean follow-up time was 14.6 ± 15.1 months. Post-operatively, 25 (34%) continued corticosteroid eye drops for more than 1 month.

**Table 1:** Pre-operative characteristics of the included patients.

**Characteristic**	**Data**
Number of patients/affected eyes, n/n	73/73
Age (years), mean (SD)	66.8 (13.3)
Sex, men/women, n (%)/n (%)	33 (45)/40 (55)
Eye, right/left, n (%)/n (%)	44 (60)/29 (40)
Anatomical type of uveitis, n (%)	
Anterior uveitis	1 (1)
Intermediate uveitis	12 (16)
Posterior uveitis	13 (18)
Panuveitis	41 (56)
Unspecified	6 (8)
Cause of uveitis, n (%)	
Vitreoretinal lymphoma	22 (30)
Acute retinal necrosis	7 (10)
Cytomegalovirus retinitis	6 (8)
Post-operative infectious endophthalmitis	4 (5)
Others	7 (10)
Unclassified	27 (37)
Lens status, n (%)	
Phakia	39 (53)
Pseudophakia	33 (45)
Aphakia	1 (1)
Best-corrected visual acuity (decimal), median (range)	0.4 (LP−1.5)
Best-corrected visual acuity (logMAR), mean (SD)	0.687 (0.802)
Intraocular pressure (mmHg), mean (SD)	15.9 (8.0)
Anterior chamber cells (grade), mean (SD)	0.6 (0.8)
Anterior chamber flare (grade), mean (SD)	0.4 (0.9)
Vitreous haze (grade), mean (SD)	1.9 (1.2)

*SD, standard deviation; logMAR, logarithm of the minimum angle of resolution; LP, light perception*.

**Figure 1:**
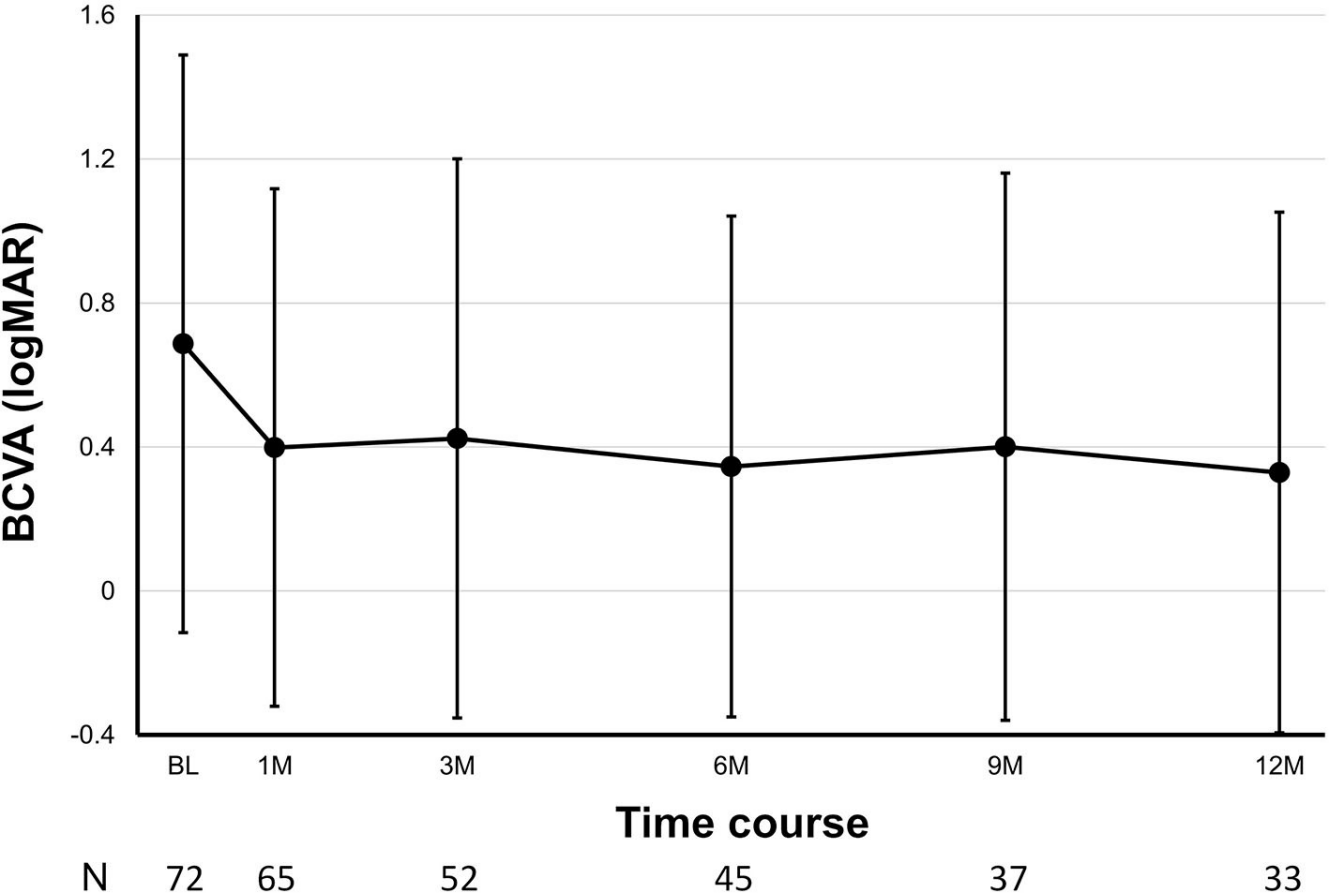
Post-operative changes in best-corrected visual acuity (BCVA) over time. Number of eyes (N) is provided at each time point.

**Figure 2:**
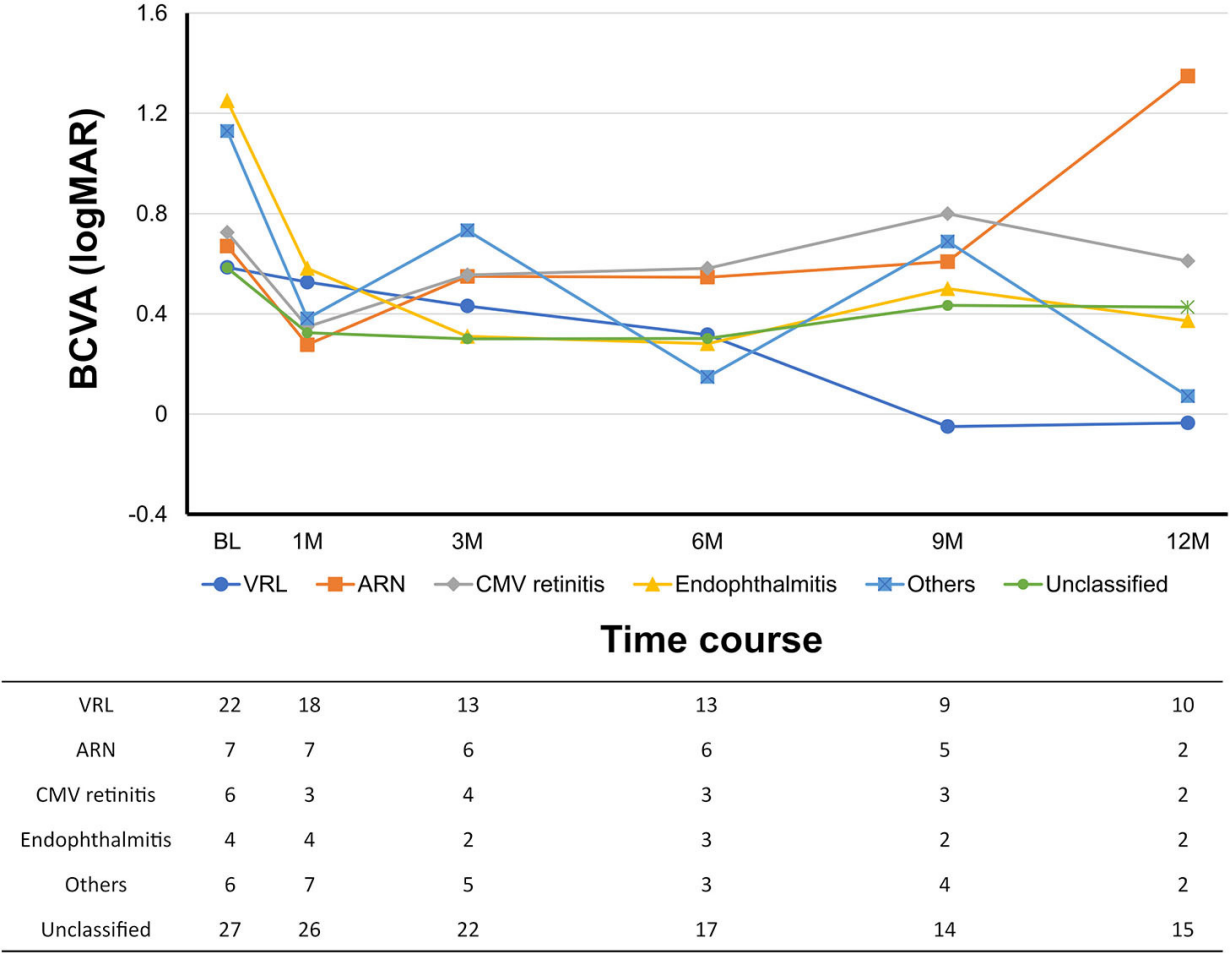
Post-operative changes in best-corrected visual acuity (BCVA) over time according to diagnosis. Number of eyes (N) is provided at each time point. VRL, vitreoretinal lymphoma; ARN, acute retinal necrosis; CMV, cytomegalovirus.

Overall, the mean logMAR BCVA was 0.69 ± 0.80 pre-operatively, 0.40 ± 0.72 at 1 month post-operatively, 0.42 ± 0.78 at 3 months post-operatively, 0.35 ± 0.70 at 6 months post-operatively, 0.40 ± 0.76 at 9 months post-operatively, and 0.33 ± 0.72 at 12 months post-operatively (*P* = 0.017, pre-operatively vs. 3 months post-operatively; Figure 1). The mean logMAR BCVA showed a significant difference at each post-operative time point as compared to pre-operatively (*P* < 0.001 at 1 month, *P* = 0.017 at 3 months, *P* = 0.021 at 6 months, *P* = 0.027 at 9 months, and *P* = 0.016 at 12 months). The percentages of eyes with “improved,” “unchanged,” and “worsened” BCVA at 3 months after surgery were 37, 50, and 13%, respectively. The percentages of eyes with a BCVA of 20/200 or better and 20/40 or better were 74 and 50% pre-operatively and 91% and 76% at 12 months post-operatively, respectively. According to diagnosis, the mean logMAR BCVA pre-operatively and at 12 months post-operatively were 0.59 ± 0.84 and −0.04 ± 0.13 in vitreoretinal lymphoma, 0.67 ± 0.89 and 1.35 ± 1.77 in acute retinal necrosis, 0.73 ± 0.89 and 0.61 ± 0.13 in cytomegalovirus retinitis, 1.25 ± 1.09 and 0.37 ± 0.46 in post-operative infectious endophthalmitis, 1.13 ± 0.96 and 0.07 ± 0.21 in others, and 0.58 ± 0.66 and 0.43 ± 0.82 in unclassified conditions, respectively (Figure 2).

**Figure 3:**
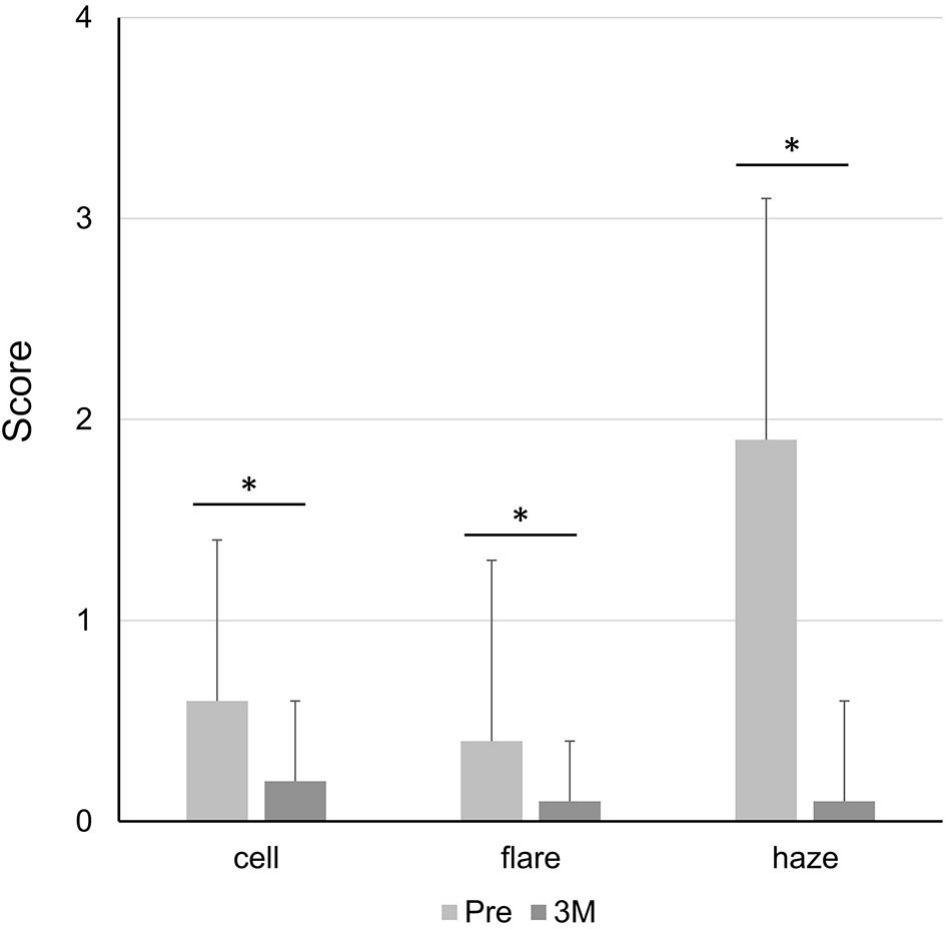
Post-operative change in inflammation scores. cell, cells in the anterior chamber; flare, flare in the anterior chamber; haze, vitreous haze; Pre, pre-operatively; 3M, at 3 months post-operatively. *P < 0.05.

The cell and flare scores in the anterior chamber as well as the vitreous haze score, significantly improved after surgery (Figure 3). The mean anterior chamber cell score was 0.6 ± 0.8 pre-operatively and 0.2 ± 0.4 at 3 months post-operatively (*P* = 0.001). The mean anterior chamber flare score was 0.4 ± 0.9 pre-operatively and 0.1 ± 0.3 at 3 months post-operatively (*P* = 0.004). The mean vitreous haze score was 1.9 ± 1.2 pre-operatively and 0.1 ± 0.5 at 3 months post-operatively (*P* < 0.001). Of the 73 eyes, 41% had 1+ or greater anterior chamber cells, 27% showed 1+ or greater anterior chamber flare, and 90% exhibited 1+ or greater vitreous haze before surgery. At 3 months post-operatively, the percentage of eyes demonstrating a 1+ or greater score decreased to 29% in anterior chamber cells, 6% in anterior chamber flare, and 4% in vitreous haze. The development of new retinal/choroidal lesions was observed in 13 (18%) eyes up to 3 months post-operatively.

Surgery-related complications occurred in 35 (48%) eyes; 6 (8%) occurred intraoperatively, 24 (33%) occurred within 2 weeks post-operatively, and 11 (15%) occurred after 2 weeks post-operatively (some cases were overlapping), as listed in Table 2. The most common intraoperative complication was retinal breaks, which were properly treated via laser photocoagulation. One eye experienced choroidal effusion due to slippage of the infusion cannula out of a sclerotomy. Ocular hypertension was the most frequent complication within 2 weeks post-operatively. Although it was transient in most cases, some eyes required glaucoma eye drops. Transient post-operative ocular hypotension also occurred during the same time frame and was often accompanied by choroidal detachment. Beyond 2 weeks post-operatively, retinal detachment was the most common complication and was successfully treated via additional vitrectomy. The incidence rate of complications between the biopsy and non-biopsy groups and among the cause of uveitis was not significant at any post-operative time point. In the comparison between eyes with and without glaucoma/ocular hypertension pre-operatively, the difference in the incidence rate of postsurgical ocular inflammation did not reach a statistical significance (*P* = 0.279).

**Table 2:** Surgical complications.

**Complication**	**Intraoperative**	**Post-operative** **(within 2 weeks)**	**Post-operative** **(after 2 weeks)**
Iatrogenic retinal break	2 (3)	0 (0)	0 (0)
Discovery of retinal tear	2 (3)	0 (0)	0 (0)
Bleeding from iris neovascularization	1 (1)	0 (0)	0 (0)
Choroid effusion	1 (1)	0 (0)	0 (0)
Ocular hypertension	0 (0)	13 (18)	2 (3)
Ocular hypotension	0 (0)	8 (11)	0 (0)
Choroidal detachment	0 (0)	4 (5)	0 (0)
Retinal detachment	0 (0)	2 (3)	4 (5)
Hyphema	0 (0)	2 (3)	0 (0)
Vitreous hemorrhage	0 (0)	1 (1)	1 (1)
Corneal erosion	0 (0)	1 (1)	0 (0)
Dislocation of intraocular lens	0 (0)	0 (0)	1 (1)
Epiretinal membrane	0 (0)	0 (0)	1 (1)
Macular edema	0 (0)	0 (0)	1 (1)
Suture-related infection	0 (0)	0 (0)	1 (1)

*Data are provided as n (%)*.

## Discussion

In the present study, we retrospectively analyzed 73 consecutive cases that underwent primary 27-gauge PPV for various vitreoretinal disorders associated with uveitis. Henry et al. conducted a systematic literature review of PPV for uveitis and summarized the data of 34 recent articles (12). The review reported the following mean or median patient demographics for each included study: number of eyes (13.5), age at the time of PPV (42.8 years), and follow-up time after PPV (19.0 months). Compared with these data, the current study had a larger number of eyes (73), older age at time of PPV (66.8 years), and similar follow-up time after PPV (14.6 months). The reason for the older age at time of PPV in our study could be attributed to the smaller number of eyes with young-onset uveitis. In Henry's review, Behçet's disease and juvenile idiopathic arthritis accounted for 8.1% and 9.3% of all eyes, respectively (12). In contrast, none of the cases in our study had Behçet's disease or juvenile idiopathic arthritis. The specified anatomical location of uveitis was similar between studies, in that ~50% of eyes had panuveitis. In the cause of uveitis, vitreoretinal lymphoma comprised 30% of all eyes, which is the greatest feature of our cohort and may affect the surgical outcomes as described below. Note that the mean surgical time of 47.1 min in our study is longer than that reported in previous studies (20.2–38.8 min for non-uveitic eyes) (17, 18, 24). This difference may be attributed to the higher rate of concomitant cataract surgery, the need to educate unexperienced nurses and assistants during surgery, or both. As surgical time in MIVS is one of the most interesting points for vitreous surgeons, further studies are required to understand the real impact of 27-gauge PPV on surgical time in eyes with uveitis.

Overall, the visual outcomes of 27-gauge PPV for uveitis in our study were promising. The mean logMAR BCVA significantly improved, with 37% of eyes attaining logMAR BCVA improvement of ≤−0.3 and 76% of eyes with a BCVA of 20/40 or better at 12 months post-operatively. The subgroup analyses based on diagnosis showed a similar tendency, with the exception of the acute retinal necrosis group wherein the mean logMAR BCVA worsened during follow-up as cases with good vision recovery returned to a local doctor. It should be noted that the method of reporting visual outcome results differs among studies, including the definition of BCVA improvement and time points of BCVA evaluation. The factors affecting post-operative visual recovery (e.g., cause of uveitis, pre-operative BCVA, and pre-operative inflammatory status) may also vary. In an analysis of 519 eyes with uveitis from 31 studies, BCVA improved in 69% of eyes, was unchanged in 18% of eyes, and worsened in 13% of eyes (12). In 25-gauge PPV for uveitis, Soheilian et al. reported that 59% of patients showed BCVA improvement (9), whereas Kamei et al. reported a mean logMAR BCVA of 0.58 at 12 weeks post-operatively (8). In the current study, 87% of eyes had improved/unchanged BCVA at 3 months post-operatively, 76% of eyes attained a BCVA of 20/40 or better at 12 months post-operatively, and the mean logMAR BCVA was 0.423 at 3 months post-operatively. Although it is not meaningful to directly compare visual outcomes among studies due to the aforementioned reasons, the visual outcomes following 27-gauge PPV in our study were generally satisfactory.

Post-operative inflammation is a concern to ophthalmologists operating on eyes with uveitis because the underlying uveitis may be exacerbated following surgery. According to a systematic literature review, intraocular inflammation generally subsides following PPV (12), which is consistent with the findings of our study. However, as most previous studies did not apply standardized grading, it is difficult to properly compare the degree of inflammation among studies. Kamei et al. reported changes in ocular inflammation in 20 eyes with uveitis that underwent 25-gauge PPV. In their report, the percentage of eyes with anterior chamber cells of 1+ or greater was 30% pre-operatively but only 5% at 12 weeks post-operatively (8). Examining data from a systematic review (12), we calculated that the percentage of eyes with anterior chamber cells of 1+ or greater was 92% pre-operatively and 52% post-operatively. In our study, the percentage of eyes with anterior chamber cells of 1+ or greater was 41% pre-operatively and 29% at 3 months post-operatively. Regarding other inflammatory signs, Kamei et al. reported that the percentage of eyes with anterior chamber flare of 1+ or greater was 25% pre-operatively and 5% at 12 weeks post-operatively, and the percentage of eyes with vitreous haze of 1+ or greater was 100% pre-operatively and 5% at 12 weeks post-operatively (8). In our study, the percentage of eyes with anterior chamber flare of 1+ or greater was 27% pre-operatively and 6% at 3 months post-operatively, and the percentage of eyes with vitreous haze of 1+ or greater was 90% pre-operatively and 4% at 3 months post-operatively. In the era of MIVS—including 27-gauge PPV—post-operative inflammation is expected to be minimal even in eyes with uveitis.

Surgery-related complications are another concern in PPV for uveitis. Although 48% of eyes experienced complications in the present study, most were mild and transient. As shown in Table 2, complications related to intraocular pressure accounted for 68% of overall complications (18% of all eyes) within post-operative 2 weeks, which is consistent with a previous report (19). Khan et al. conducted a multicenter retrospective study of 360 patients who underwent 27-gauge PPV for posterior segment disease (not limited to uveitis) and found that 16% of eyes showed ocular hypertension or hypotony post-operatively (19). The extent of intraoperative and post-operative complications in PPV for uveitis is best summarized by Henry et al. (12). After reviewing 34 articles published from 2005 to 2014 and analyzing 708 eyes (~25% of which were treated with 25-gauge PPV and no cases treated with 27-gauge PPV), they reported the following frequencies of surgery-related complications: cataract (24%), glaucoma (17%), epiretinal membrane (15%), band keratopathy/corneal decompression (11%), pupillary block (9.3%), etc. The rates of complications in Henry et al.'s review are obviously more severe compared with those reported in our study, suggesting that the 27-gauge system contributes to safer vitreous surgery in uveitis eyes.

This study has some limitations inherent to its retrospective nature. First, the surgical technique was generally standardized, but the use of adjunctive procedures (e.g., concomitant cataract surgery, intraoperative triamcinolone acetonide injection, suture placement at sclerotomies, etc.) was performed at the surgeon's discretion and may have affected surgical outcomes. Second, we included various types of surgical indications. Although it might help to understand the overall picture of surgical outcomes following 27-gauge PPV for uveitis, the number of eyes by diagnosis or by purpose of surgery was insufficient to perform detailed analyses. Third, because we only included subjects having eyes treated with primary 27-gauge PPV, no data were provided on 27-gauge PPV for previously vitrectomized eyes or inter-eye comparison for patients who underwent 27-gauge PPV in both eyes. Further well-designed clinical studies are warranted to address these concerns.

In conclusion, we have reported the clinical outcomes of primary 27-gauge PPV for various vitreoretinal disorders associated with uveitis. In 27-gauge PPV for uveitis, BCVA was maintained or improved in most cases, whereas intraocular inflammation typically subsided post-operatively. Although nearly half of the patients experienced surgery-related complications, they tended to be mild and transient. Given its risk–benefit profile, 27-gauge PPV is a promising option for the treatment of vitreoretinal disorders in uveitis.

## Data Availability Statement

The raw data supporting the conclusions of this article will be made available by the authors, without undue reservation.

## Ethics Statement

The studies involving human participants were reviewed and approved by the Institutional Review Board of the Kobe University Graduate School of Medicine. Written informed consent for participation was not required for this study in accordance with the national legislation and the institutional requirements.

## Author Contributions

KK, SK, and WM conceived and designed the study. KK, NS, and RN contributed to data acquisition. HI, WM, and MN provided critical advice for the revision of the manuscript. SK had full access to all data in the study and takes responsibility for the integrity of the data and accuracy of the data analysis. All authors participated in data interpretation and approved the final version for publication.

## Conflict of Interest

The authors declare that the research was conducted in the absence of any commercial or financial relationships that could be construed as a potential conflict of interest.

## Publisher's Note

All claims expressed in this article are solely those of the authors and do not necessarily represent those of their affiliated organizations, or those of the publisher, the editors and the reviewers. Any product that may be evaluated in this article, or claim that may be made by its manufacturer, is not guaranteed or endorsed by the publisher.
